# The effect of light therapy on sleep quality in cancer patients: a systematic review and meta-analysis of randomized controlled trials

**DOI:** 10.3389/fpsyt.2023.1211561

**Published:** 2023-07-10

**Authors:** Liqing Yao, Zhiyi Zhang, Lawrence T. Lam

**Affiliations:** ^1^Faculty of Medicine, Macau University of Science and Technology, Taipa, Macao SAR, China; ^2^Faculty of Health Sciences, University of Macau, Taipa, Macao SAR, China; ^3^Faculty of Health, The University of Technology Sydney, Sydney, NSW, Australia; ^4^Faculty of Medicine and Health, University of Sydney, Sydney, NSW, Australia

**Keywords:** light therapy, sleep quality, sleep problems, cancer, systematic review, meta-analysis

## Abstract

**Background:**

Sleep problem is one of the major issues of cancer patients and may have detrimental effects on the ongoing treatment and recovery of patients. However, the evidence for the effect of light therapy on sleep problems in this population remained scarce. This study aimed to examine the effect of light therapy on self-reported and physiological measures of sleep quality of cancer patients. It also aimed to quantify the magnitude of the effect using a meta-analytical approach.

**Methods:**

Six databases were searched for randomized control trials (RCTs). The primary outcome was the sleep quality using the Pittsburgh sleep quality index (PSQI) measurement of self-reported scores, and the secondary outcomes included total sleep time and sleep efficiency measured by actigraphy. Meta-analyses were performed with the random effects model using the RevMan software. The standardized mean difference (SMD) of the PSQI scores and other measures with their 95% confidence intervals (CIs) were used for assessing the treatment effect (CRD42023370947).

**Results:**

Nine RCTs were identified and included in the study. Light therapy significantly improved the self-reported sleep quality with a reduction of the pooled PSQI score (SMD = −0.72; 95% CI: −1.24 to −0.21; *p* = 0.006). Regarding total sleep time (*p* = 0.72) and sleep efficiency (*p* = 0.47), no significant effects of light therapy were found.

**Conclusion:**

Light therapy could improve self-reported sleep quality in cancer patients. However, due to the heterogeneity and small sample size of the included trials, the results should be interpreted cautiously. Trials with better designs and larger sample sizes are suggested to be conducted for a more definitive conclusion.

**Systematic review registration:**https://www.crd.york.ac.uk/PROSPERO/display_record.php?RecordID=370947.

## 1. Introduction

Cancer is one of the main causes of death, with mortality of nearly 10 million accounting for one in six deaths in 2020 worldwide (1). During the progression and the treatments of cancer, patients may have experienced other co-morbidities, such as emotional disorders (including anxiety and depression), fatigue, and sleep disorders (2–4). It has been shown that nearly 60% of cancer patients have suffered from sleep problems, such as somnolence, short duration of nighttime sleep, poor sleep quality, and difficulty falling asleep (5). Previous reports have also indicated that many patients may suffer from sleep problems for at least 6 months or longer (6). Sleep problems among patients with different advanced cancers have been reported to be more severe with a prevalence of 72% (7). Sleep disorders, if undiagnosed and untreated, could be conducive to severe mental and physical problems in cancer patients (8). For example, a previous study showed that sleep problems were bidirectionally associated with depression, and sleep problems could also cause serious cardiovascular problems and reduced immunity (9). These adverse effects of sleep problems on the overall health of cancer patients could be exacerbated due to their compromised health condition. This, in turn, adds a burden to the treatment, management, and recovery of patients.

Sleep disturbances and different sleep disorders (e.g., insomnia, sleep-related breathing disorder, and obstructive sleep apnea syndrome) are common and considerable complaints of cancer patients. Pharmacological therapies (e.g., melatonin, mirtazapine, and valerian herbal extracts) (10–12) and nonpharmacological therapies [e.g., psychoeducational intervention and cognitive behavior therapy (CBT)] are conducted to deal with sleep problems (13). Pharmacological therapies for sleep disorders are available and effective, however, the side effects of these medications may be generated. These include psychomotor and cognitive problems, drowsiness, poor judgment, drug dependence, and tolerance (14, 15). Due to the side effects of pharmacological therapies, non-pharmacological treatment options have been advocated with the benefits of having fewer side effects and being more cost-effective (4, 16–18).

Compared with other non-pharmacological therapies, light therapy has been gaining attention because it is critical to all forms of life, and the circadian rhythms of human beings and other species are strongly affected by light (19). Biologically, the suprachiasmatic nucleus of (SCN) hypothalamic system generates circadian rhythms through sensing the light and other stimuli in animals including humans (20). Based on this biological fact, light therapy has been developed as a treatment option for sleep disorders. It was proposed that light therapy other than natural light could retrain the circadian rhythms by triggering the SCN in sleep disorder patients (20–23). A previous study also showed that light therapy influenced the sleep/wake cycle by its action on the inhibition of melatonin and its alerting effects on the ascending arousal system (24). Normal secretion of melatonin is the key factor in maintaining the regular sleep/wake cycle. Moreover, the secretion of melatonin is regulated by the SCN in response to light signals received directly through the retinohypothalamic tract (20). Therefore, the circadian rhythm of melatonin secretion is considered the best peripheral estimator of the timing of the internal circadian pacemaker, which plays an essential role in human sleep (20, 25). Previous studies also showed that light therapy had clinical effects on depression (26–28), which can indirectly improve the sleep quality of cancer patients. Hence, the biological and psychological mechanisms provide the basis for light therapy to be considered a possible option for the treatment of sleep problems. In terms of the application of light therapy, some devices have been designed and used in clinical treatment. These include light boxes and light glasses which can emit varied types and different intensities of light (21). The advantages of light therapy, including its safety, low cost, and easy to handle, are the reasons for it to be advocated (21, 29).

In terms of the effectiveness of light therapy as a treatment option, there have been an increasing number of clinical trials investigating the effect of light therapy on sleep problems in patients with cancers (18, 24, 30). Several studies have shown that bright light therapy relieved fatigue, improved self-reported sleep quality, and reduced insomnia symptoms of post-treatment in cancer survivors (18, 31, 32). However, the strength of evidence provided by these studies is still inadequate and further research is required to further confirm the clinical effectiveness of light therapy for sleep problems in cancer patients (18, 31, 32). Currently, there are very few studies examining the magnitude of the treatment effect to provide an accurate estimation as evidence for the application of light therapy for sleep problems. Hence, this systematic review and meta-analysis aimed to examine the treatment effect of light therapy on the self-reported and physiological measures of sleep quality of cancer patients.

## 2. Materials and methods

### 2.1. Studies search and selection based on inclusion and exclusion criteria

This study was conducted based on the Preferred Reporting Items for Systematic Reviews and Meta-Analyses (PRISMA) guidelines (33). The study protocol was registered at the International Prospective Register of Systematic Reviews (PROSPERO, registration number: CRD42023370947).

**Figure 1:**
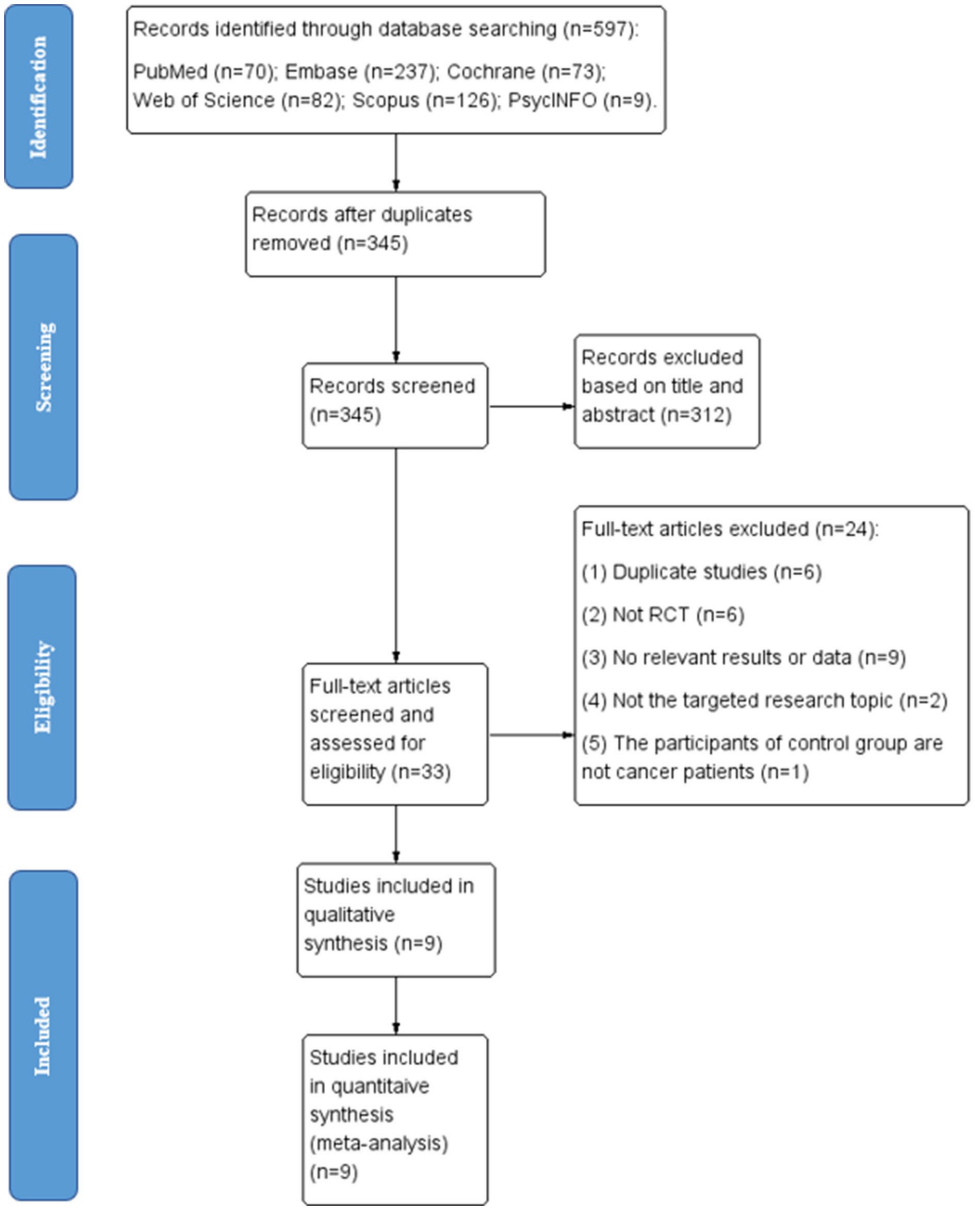
Study flow diagram based on Preferred Reporting Items for Systematic Reviews and Meta-Analyses (PRISMA) statement.

According to the PRISMA guidelines, the flow diagram of the study selection process, including the literature search, the reasons for exclusions, and the number of included studies was depicted (see Figure 1). Two authors (LQY and ZYZ) conducted the database search. The systematic search yielded 597 studies (70 from PubMed, 237 from Embase, 73 from Cochrane, 82 from Web of Science, 126 from Scopus, and 9 from PsycINFO) between January 1970 and November 2022. Medical Subject Headings (MeSH) and free terms were combined in the process of the systematic search, using the following MeSH terms: phototherapy, neoplasms, and sleep quality. More detailed search strategies were presented in Supplementary Table S1 as supporting materials.

The same two authors (LQY and ZYZ) independently screened all selected titles and abstracts, and read the full texts of relevant articles for eligibility. The eligibility criteria for inclusion were: (1) The studies are randomized controlled trials (RCTs) being published in English; (2) The participants were cancer patients or survivors; (3) Intervention measures were specifically bright light therapies, while the control measures were dim light therapies, placebo or usual care; (4) The primary outcomes were measured by the Pittsburgh Sleep Quality Index (PSQI); the secondary outcomes, namely the total sleep time and sleep efficiency at the end of the intervention, were assessed using physiological sleep measurement devices (e.g., sleep wrist actigraphy). Studies were excluded if they were: (1) not RCTs; (2) duplicated studies; (3) missing data on the primary and secondary outcomes; (4) not published in English; (5) the controls were not cancer patients; (6) the intervention measures included a combination of light therapy with another treatment (e.g., cognitive behavior therapy), while the control group did not receive the same extra treatment. For duplicated studies, data were only extracted from the one with the most complete and updated information.

### 2.2. Data extraction and assessment for risk of bias

Data were individually extracted and checked by authors YLQ and ZYZ independently using a predesigned data extraction form designed with Microsoft Excel. The following data of study characteristics were extracted: author, year of publication, country, sample size, age, gender, type of cancer, stage of cancer, the timeframe of diagnosis or therapy, previous treatment, intervention, and control descriptions, intervention duration, primary and secondary outcomes. The risk of bias was independently evaluated according to the Cochrane risk-of-bias assessment tool (34). Selection bias, performance bias, detection bias, attrition bias, and reporting bias were included as assessment items. The risk of bias was classified as low, high, or unclear. Any discrepancies were resolved by consensus or discussions with the third author (LTL).

### 2.3. Data synthesis and statistical analysis

Review Manager software (RevMan version 5.3) was used to perform the meta-analysis. The mean and standard deviations (SDs) of the outcomes at the trial were extracted and estimated. If the studies did not report mean and SDs, the authors of the trials were contacted via email to request the information. According to Cochrane Handbook Version 6.3 and the previous study (35, 36), if it was possible to calculate mean and SDs from other data provided in the included studies (e.g., standard error (SE), *p* values, or t values), it would be performed. If the information was unavailable to assess the mean and SDs, the studies were excluded from the meta-analysis, although they had been included in the systematic review. The effect sizes were presented as the standardized mean differences (SMDs) between the intervention group with the light therapy and control groups with the corresponding 95% confidence intervals (CIs). Cohen’s classifications were used to categorize the effect sizes (SMD 0.2–0.5 = small effect, SMD 0.6–0.8 = moderate effect, and SMD > 0.8 = large effect) (37). In this study, a negative SMD indicated an improvement in sleep quality outcomes. Heterogeneity was assessed by using the Cochran’s Q test and I^2^ statistic. In general, *p* < 0.10 in the Cochran’s *Q* test or *I*^2^ > 50% indicated a substantially significant heterogeneity. More specifically, 0–25% suggested that the heterogeneity issue is insignificant, 26–50% represented a low heterogeneity issue, 51–75% moderate, and 76–100% high heterogeneity (38). The random effects model was used in the meta-analysis for effect estimations, based on the assumption that the sample used is one of the many selected from the population. To explore the source of any heterogeneity issues, subgroup analyses were conducted, based on different characteristics of the studies, to examine for any differences in the effect estimates. Furthermore, sensitivity analyses involving deleting each study one by one were also carried out to investigate the source of heterogeneity and assess the robustness of the pooled estimates.

## 3. Results

### 3.1. Characteristics of included studies

Nine studies in total met all eligibility criteria and were included in the meta-analysis. These studies included a total of 451 participants, with 234 in the intervention group and 217 in the control group. Of these, five were conducted in the US (24, 30, 31, 39, 40), two in Turkey (41, 42), one in Canada (43), and another one in the Netherlands (44). The average age of the participants in these studies was 55.32 years. Participants in five RCTs were patients with specific types of cancers including breast cancer, lung cancer, ovarian, endometrial cancer, Hodgkin lymphoma (HL), and diffuse large B-cell lymphoma (DLBCL) (31, 39–41, 44). The rest included patients with nonspecific cancers (24, 30, 42, 43). These participants had previously received basic treatment such as radiotherapy and chemotherapy. In terms of the color of the lights, white, green, green-blue, or white-blue were used in the intervention groups, while the dim red/white lights or usual care were performed in the control groups. The light intensities ranged from 417.9 lux to 10,000 lux, and the intervention duration was 1, 2, 3.5, 4, and 8–12 weeks with the exposure time ranging between 30 and 60 min in the morning or afternoon. Characteristics of the included literatures of this study are presented in Table 1.

**Table 1:** Characteristics of included studies.

Author (Year)	Country	Participants		Type of cancer	Timeframe of diagnosis or therapy	Treatment	Duration	Outcome	
		Intervention	Control					Primary outcome	Secondary outcome
Ozkaraman et al. (2018)	Turkey	Number: 11 Age: 53.36 ± 2.35 Gender: *F* = 11	Number: 12 Age: 49.25 ± 3.33 Gender: *F* = 12	Breast cancer	Undergoing radiotherapy	I: 2000–3000 lux bright white light; C: Daily radiotherapy session.	30 min/d before radiotherapy sessions in the afternoon, 1 week	PSQI	NA
Weiss et al. (2018)	United States	Number: 7 Age: 68.8 ± 7.2 Gender: *F* = 5/M = 2	Number: 5 Age: 66.0 ± 10.1 Gender: *F* = 1/M = 4	Lung cancer	Completed treatment at least 6 weeks and no longer than 3 years	I: 417.9 lux green-blue light (500 nm), light glasses; C: 152.3 lux red-yellow light.	60 min/d in the morning for 60 min within 1 h upon awakening, 1 week	PSQI	NA
Wu L. M. et al. (2018)	United States	Number:25 Age: 53.0 ± 12.1 Gender: *F* = 20/M = 5	Number: 19 Age: 54.1 ± 9.4 Gender: F = 13/M = 6	Hematological malignancy, breast cancer, gynecological cancer	Time since primary treatment: 1.04 ± 0.72 years (intervention), 1.60 ± 0.82 years (control)	I: 1,350 lux full spectrum white light, Litebook; C: <50 lux dim red light	30 min/d every morning, 4 weeks	PSQI	Actigraphy: total sleep time/min, sleep efficiency/%
Yennurajalingam et al. (2020)	United States	Number:8 Age: NA Gender: NA	Number: 8 Age: NA Gender: NA	Advanced cancer	Undergoing treatment	I: 1350 lux white light, the Litebook; C: 50 lux dim red light.	30 min each morning within 2 h of arising before noon, 2 weeks	PSQI	NA
Garland et al. (2020)	Canada	Number: 42 Age: 56.57 ± 10.49 Gender: *F* = 38/M = 4	Number: 39 Age: 59.97 ± 9.26 Gender: *F* = 32/M = 7	Cancer	Completed treatment at least 3 months	I: 1250 lx white-blue light (∼465 nm), light-emitting diodes (LEDs); C: <400 lx red light (∼633 nm)	30 min/d every morning, 4 weeks	PSQI	Actigraphy: total sleep time/min, sleep efficiency/%
Fox et al. (2020)	United States	Number: 9 Age: 53.89 ± 11.20 Gender: *F* = 9	Number: 9 Age: 60.33 ± 7.94 Gender: *F* = 9	Ovarian and endometrial cancer	Had no history of chemotherapy or had completed primary chemotherapy at least 30 days	I: 506 lx lm/m2 bright light, LEDs; C: Dim red light or green light.	45 min/d or at least 30 min/d every morning, 4 weeks	PSQI	Wrist actigraphy: total sleep time/min, sleep efficiency/%
Starreveld et al. (2021)	The Netherlands	Number: 83 Age: 46.7 ± 11.9 Gender: *F* = 50/M = 33	Number: 83 Age: 44.8 ± 12.5 Gender: *F* = 49/M = 34	Hodgkin lymphoma (HL) and diffuse large B-cell lymphoma (DLBCL)	The time since diagnosis of all survivors was 12.9 ± 9.9 years	I: 1500 lux bright white light (468 and 570 nm), Luminette glasses; C: 8 lux dim white light (468 and 570 nm).	30 min/d after waking, 25 days	PSQI	Sleep wrist actigraphy: total bedtime (total sleep time)/min, sleep efficiency/%
Celik et al. (2022)	Turkey	Number: 26 Age: NA Gender: *F* = 13/M = 13	Number: 26 Age: NA Gender: *F* = 13/M = 13	Breast cancer, gynecological cancer, gastrointestinal cancer, lung cancer, head and neck cancer and urological cancer	The total diagnosis time: <23 months (*n* = 26), >23 months (*n* = 26)	I: 10,000 lux bright white light, a specially designed lightbox; C: <50 lux dim red light.	30 min every morning, 2 weeks	PSQI	Smart wristbands: total sleep time/min
Rissling et al. (2022)	United States	Number: 23 Age: 54.26 ± 9.31 Gender: *F* = 23	Number: 16 Age: 53.50 ± 8.96 Gender: *F* = 16	Breast cancer	Undergoing chemotherapy	I: 1500 lux white light, Litebook; C: <50 lux dim red light.	30 min upon awakening every day, 8–12 weeks	PSQI	Actigraphy: nighttime total sleep time/min and sleep percentage (sleep efficiency)/%

I, Intervention group; C, Control group; F, Female; M, Male; NA, Not available; PSQI, Pittsburgh Sleep Quality Index.

### 3.2. The effect of light therapy on sleep quality in different sleep indexes

Nine studies were included in the meta-analysis reporting PSQI as the primary outcome (24, 30, 31, 39–44). The results revealed that the average PSQI scores of the light therapy group were significantly lower than that of the control group (SMD = −0.72; 95% CI: −1.24 to −0.21; *p* = 0.006), suggesting light therapy might improve the overall sleep quality in cancer patients. However, the level of heterogeneity in the overall analysis was high (*p* < 0.00001; *I*^2^ = 80%), suggesting a considerable amount of variabilities in these studies.

Six studies were included in the meta-analysis reporting total sleep time (30, 31, 40, 42–44). The pooled results showed that there were no significant differences between groups (SMD = 0.04; 95% CI: −0.20 to 0.29; *p* = 0.72), with insignificant heterogeneity issues (*p* = 0.32; *I*^2^ = 14%).

Five studies were included in the meta-analysis reporting sleep efficiency (30, 31, 40, 43, 44). The pooled results showed that light therapy had no significant effect on sleep efficiency (SMD = 0.11; 95% CI: −0.19 to 0.41; *p* = 0.47). The results indicated that the heterogeneity was low (*p* = 0.24; *I*^2^ = 27%).

**Figure 2:**
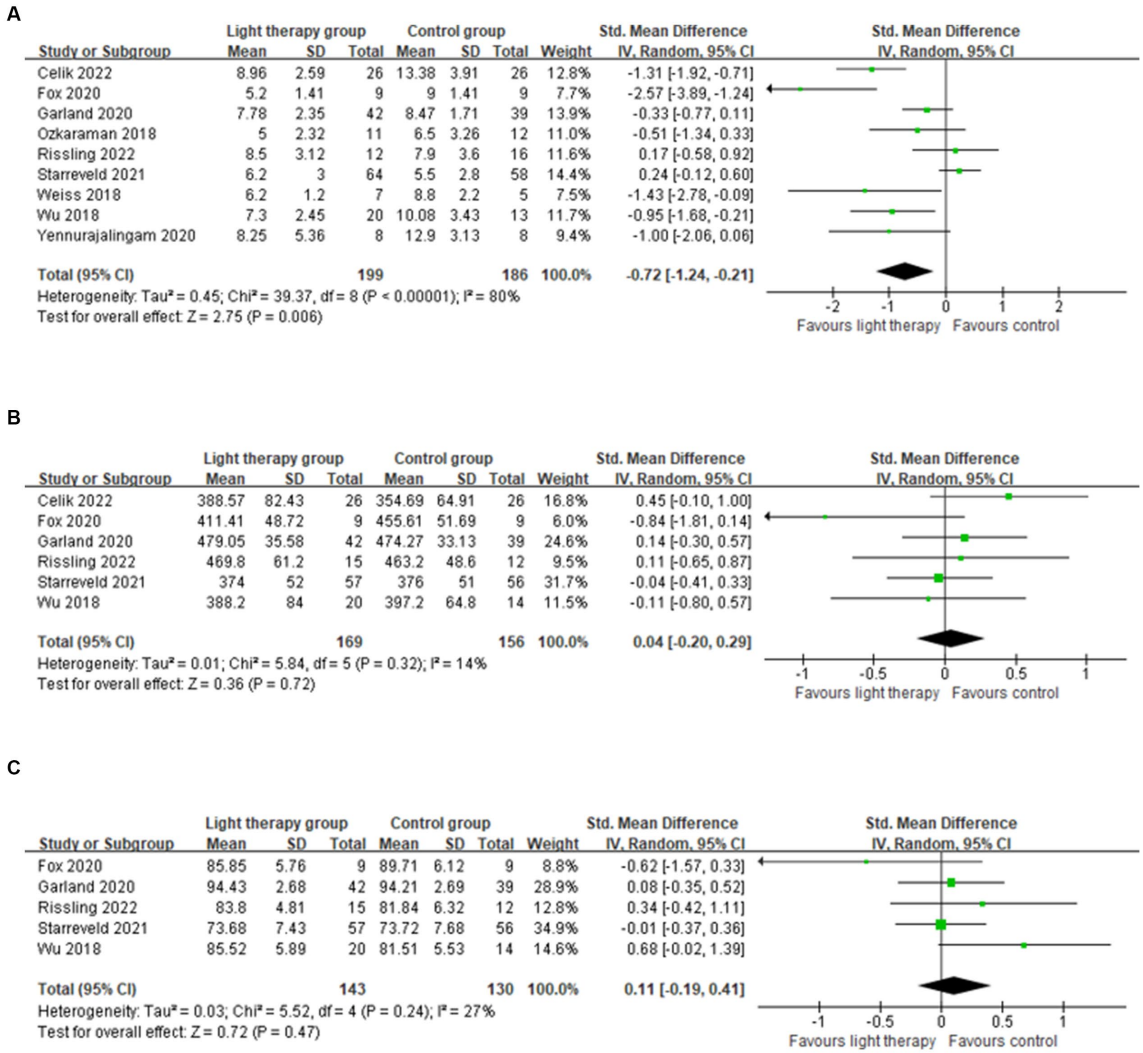
Forest plot of light therapy on self-reported instrument and physiological sleep quality indexes. **(A)** Forest plot of light therapy on PSQI. **(B)** Forest plot of light therapy on total sleep time (min). **(C)** Forest plot of light therapy on sleep efficiency (%).

The forest plots of light therapy on PSQI and physiological sleep quality indexes were presented in Figure 2.

### 3.3. Risk of bias in the included studies

**Figure 3:**
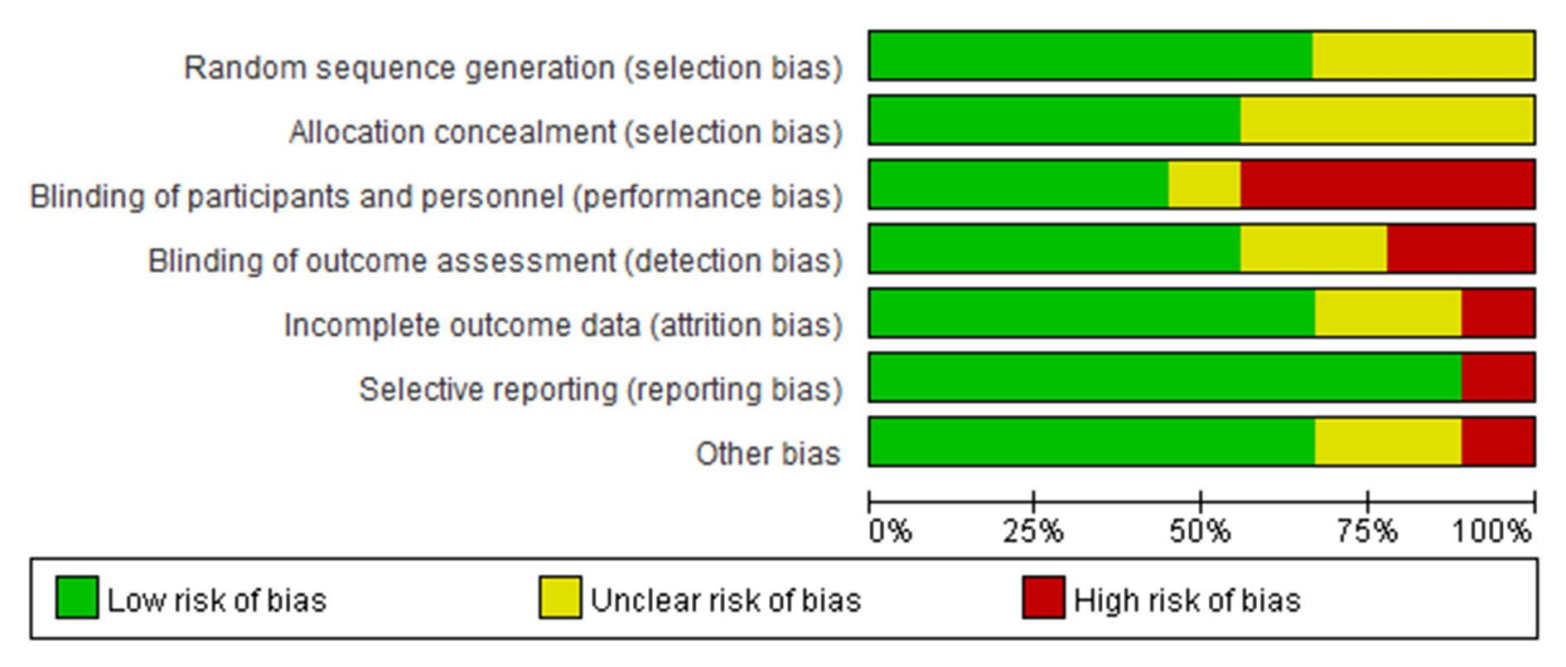
Risk of bias graph: review authors’ judgments about each risk of bias item presented as percentages across all included studies.

**Figure 4:**
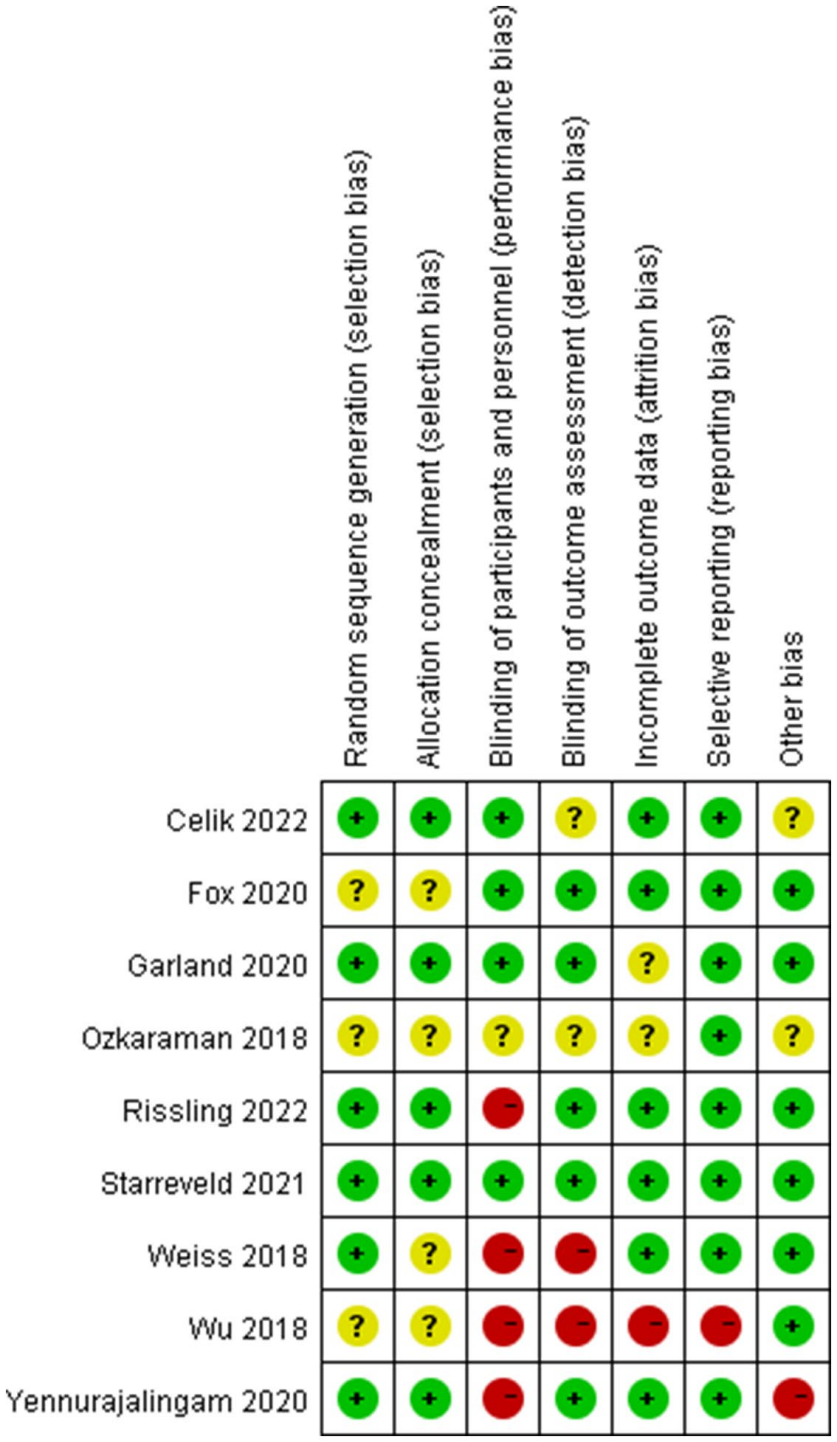
Risk of bias summary: review authors’ judgments about each risk of bias item for each included study.

The results of the risk of bias assessment were summarized in Figures 3, 4. As shown, performance bias was the most common type of bias in these studies. Of the nine studies included in the systematic review, five had issues with blinding with four having definite and one possible risk (24, 30, 31, 39, 41). Given the design of these studies, it was likely that the blinding issue was more related to research personnel than the participants. The second most common bias noted was the detection bias which related to the blinding of the outcome assessment. Nearly half of these studies (four of the nine) had been identified with an issue of blinded outcome assessment (30, 39, 41, 42). Three studies were found to have a problem of attrition with incomplete outcome assessments (30, 41, 43). There were also other biases involved in these studies. For example, three failed to report the details of random sequence generation (30, 40, 41); four did not mention the process of allocation concealment (30, 39–41); one was high-risk of bias because this study was a factorial design which used two of eight arms (24). Of the nine studies, one had a considerable risk of bias with four on high and two on moderate of the seven risk item (30).

### 3.4. Subgroup analyses

The study’s authors further conducted subgroup analyses for PSQI, total sleep time, and sleep efficiency based on the following variables: country, gender, the number of participants, and light duration. Regarding the country, the effect size for PSQI showed a significant difference between the intervention group with the light therapy and control groups in the US (SMD = −1.05; 95% CI: −1.89 to −0.21; *p* = 0.01; *n* = 5) (24, 30, 31, 39, 40), while there was no significant difference among the studies conducted in other countries (SMD = −0.45; 95% CI: −1.11 to 0.22; *p* = 0.19; *n* = 4) (41–44). As for the number of participants (< 50 versus ≥50), the results showed that the studies involving less than 50 participants had a significant reduction in the PSQI scores (SMD = −0.93; 95% CI: −1.60 to −0.25; *p* < 0.01; *n* = 6) (24, 30, 31, 39–41), while there was no significant difference among the studies involving more than 50 participants (SMD = −0.44; 95% CI: −1.25 to 0.38; *p* = 0.29; *n* = 3) (42–44). Regarding the light duration of the light intervention (< 4 weeks versus ≥4 weeks), as for the pooled effect size of PSQI scores, the studies of <4 weeks and the studies of ≥4 weeks both showed no significant differences between light therapy groups and control groups (all *p* > 0.05). As for total sleep time and sleep efficiency, the pooled effect size for the subgroups of all variables showed no significant differences between light therapy groups and control groups (all *p* > 0.05), and no significant subgroup differences of enhancing effects were found in total sleep time and sleep efficiency (all *p* > 0.05). The results of subgroup analyses were presented in Table 2.

**Table 2:** Subgroup analyses of all sleep indexes.

Sleep indexs	Variable	No. of studies	Sample size	SMD (95%CI)	Test for overall effect		Heterogeneity		*p* value for subgroup difference
					Z	*p* value	I^2^	*p* value	
PSQI	Country								0.27
	US	5	107	−1.05 (−1.89, −0.21)	2.44	0.01	72%	<0.01	
	Other countries	4	278	−0.45 (−1.11, 0.22)	1.32	0.19	85%	<0.01	
	The number of participants								0.37
	<50	6	130	−0.93 (−1.60, −0.25)	2.69	<0.01	66%	0.01	
	≥50	3	255	−0.44 (−1.25, 0.38)	1.05	0.29	90%	<0.01	
	Duration								0.97
	<4 weeks	5	225	−0.73 (−1.53, 0.07)	1.79	0.07	84%	<0.01	
	≥4 weeks	4	160	−0.76 (−1.57, 0.06)	1.82	0.07	79%	<0.01	
Total sleep time	Country								0.26
	US	3	79	−0.20 (−0.69, 0.29)	0.79	0.43	15%	0.31	
	Other countries	3	246	0.12 (−0.13, 0.38)	0.94	0.35	4%	0.35	
	The number of participants								0.26
	<50	3	79	−0.20 (−0.69, 0.29)	0.79	0.43	15%	0.31	
	≥50	3	246	0.12 (−0.13, 0.38)	0.94	0.35	4%	0.35	
	Duration								0.5
	<4 weeks	2	165	0.16 (−0.31, 0.63)	0.67	0.50	52%	0.15	
	≥4 weeks	4	160	−0.04 (−0.38, 0.31)	0.22	0.83	12%	0.33	
Sleep efficiency	Country								0.67
	US	3	79	0.20 (−0.51, 0.90)	0.54	0.59	57%	0.10	
	Other countries	2	194	0.03 (−0.25, 0.31)	0.21	0.83	0%	0.77	
	The number of participants								0.67
	<50	3	79	0.20 (−0.51, 0.90)	0.54	0.59	57%	0.10	
	≥50	2	194	0.03 (−0.25, 0.31)	0.21	0.83	0%	0.77	
	Duration								0.55
	<4 weeks	1	113	−0.01 (−0.37, 0.36)	0.03	0.98	NA	NA	
	≥4 weeks	4	160	0.17 (−0.27, 0.61)	0.76	0.45	40%	0.17	

NA, Not available.

### 3.5. Sensitivity analysis and publication bias

Due to the high heterogeneity in PSQI outcomes, sensitivity analysis was conducted by deleting each study to estimate the effect of the individual study on the final results. Consistent results were yielded in most of the outcomes. However, when the study conducted by Starreveld was excluded, the *I*^2^ value of heterogeneity decreased slightly from 80 to 68%, however, the *p*-value was significant (44). As the number of included studies in our meta-analysis was less than 10, funnel plots and the Egger’s test were not performed to measure publication bias. The results of the sensitivity analysis were presented in Table 3.

**Table 3:** Sensitivity analysis of PSQI scores.

Deletion	Heterogeneity	SMD; 95%CI; *p*-value
Celik 2022	*p* < 0.001; *I*^2^ = 76%	SMD-0.62; 95%CI (−1.14, −0.10); *p* = 0.02
Fox 2020	*p* < 0.001; *I*^2^ = 76%	SMD-0.56; 95%CI (−1.03, −0.08); *p* = 0.02
Garland 2020	*p* < 0.001; *I*^2^ = 82%	SMD-0.82; 95%CI (−1.45, −0.18); *p* = 0.01
Ozkaraman 2018	*p* < 0.001; *I*^2^ = 82%	SMD-0.76; 95%CI (−1.34, −0.19); *p* < 0.01
Rissling 2022	*p* < 0.001; *I*^2^ = 81%	SMD-0.85; 95%CI (−1.41, −0.28); *p* < 0.01
Starreveld 2021	*p* = 0.003; *I*^2^ = 68%	SMD-0.86; 95%CI (−1.36, −0.37); *p* < 0.01
Weiss 2018	*p* < 0.001; *I*^2^ = 81%	SMD-0.67; 95%CI (−1.20, −0.13); *p* = 0.01
Wu 2018	*p* < 0.001; *I*^2^ = 81%	SMD-0.70; 95%CI (−1.27, −0.14); *p* = 0.01
Yennurajalingam 2020	*p* < 0.001; *I*^2^ = 82%	SMD-0.70; 95%CI (−1.25, −0.15); *p* = 0.01

## 4. Discussion

To our knowledge, this is the first systematic review and meta-analytical study on the effect of light therapy in improving sleep quality including both self-reported and physiological measures of the sleep quality outcomes in cancer patients. The findings of this systematic review and meta-analysis are in line with the results obtained from other reviews that light therapy had a beneficial effect on the self-perceived sleep quality in cancer patients (29, 45). On the other hand, there were no significant differences between the intervention group with the light therapy and control groups in terms of total sleep time and sleep efficiency. Recent systematic review studies on light therapy as an interventional therapeutic approach had also been conducted in cancer patients (29, 45). While the main outcome measures of one of these two studies were patient fatigue and depression, it also included meta-analytical results on sleep disturbance as assessed by PSQI (29). The results obtained in the Xiao et al. (29) study suggested a significant reduction in the overall PSQI score in favor of the light therapy group. The results obtained from the current study are consistent with that reported in the literature. Moreover, the current review and meta-analyses have covered, not only the self-reported measure of sleep quality but also actigraphy information collected from the wearable devices. This could be considered an extension of the existing literature in contributing to the pool of knowledge.

It is worth noting that there were a considerable amount of variabilities in the included studies as reflected in the test of heterogeneity with an *I*^2^ value of about 80% for the meta-analysis of the primary outcome PSQI. These variabilities could have been related to the included studies with a range of sample sizes, particularly with three very small studies with less than 10 in each arm. However, such an argument might not be supported by the results obtained from the subgroup analyses with sample sizes <50 and ≥ 50 as shown in Table 2. As shown, studies with smaller sizes provided a significant result and a smaller *I*^2^ value, in comparison to the larger size studies. Moreover, further sensitivity analyses revealed little changes in the overall results and the test of heterogeneity results between the full sample (i.e., nine studies included) and the trimmed sample (with three small-sized studies removed). Hence, the sample size might not be the main reason, and there would be other sources of variabilities, such as the clinical characteristics of different samples. This is worthy of further research in the future. The results obtained on the risk of biases analyses suggested a heterogeneity issue with the outcome of PSQI scores. In addition, only a small number of trials were found on the topic and the sample sizes of most of these trials were small. This might render the meta-analyses lacking the power to demonstrate a true effect. Hence, these results should be interpreted with caution. As aforementioned, as a treatment option, light therapy could be considered complementary to the current pharmacological management of sleep problems in cancer patients (24, 31, 39, 46).

In terms of the possible biological mechanism for light therapy as a treatment option for sleep problems, particularly among cancer patients, had been briefly described above in the previous section. In brief, light therapy performed at specific periods during the day may stimulate the SCN and suppress the release of the sleep hormone melatonin (21). Therefore, light therapy may increase the cancer patient’s activity during the day and reduce it at night to regulate individual circadian rhythm, and subsequently improves sleep quality.

There are strengths as well as limitations in the current meta-analysis. First, this study is one of the few to quantitatively evaluate the effect of light therapy on improving sleep quality in cancer patients. It has evaluated multiple dimensions of sleep quality including self-reported and physiological measures of sleep outcomes. Second, this is a study that includes RCTs only aiming to elicit the best evidence from studies of a better design in terms of strength of evidence. Third, the outcome measures, particularly the physiological sleep quality outcomes of total sleep time and sleep efficiency, were measured by using actigraphy in the included studies. Although the use of the actigraphy method for data collection may offer some objective measures of the outcome, however, due to the lack of comparisons among different types and brands of actigraphy equipment for accuracy and sensitivity, there could also be bias introduced. The use of actigraphy for the assessment of sleep quality is considered a better choice for physiological measures in sleep studies (47). Some limitations have also been identified in this study. First, the heterogeneity issue of the meta-analysis suggested a high degree of variability in the included studies. There could be many reasons for such an observation. One may be due to the differences in various intervention characteristics of light therapy. Second, there were only 9 randomized controlled trials included with limited sample sizes, which might have caused an overall underpower of the meta-analysis, and in turn, caused a type II error. Third, subgroup analyses and sensitivity analyses had been conducted to explain this heterogeneity but there remained substantial or high heterogeneity in most of the results. Other underlying variables such as types of cancer, cancer stage, previous treatments, phototherapy intensities and devices and therapeutic environment might account for this. However, due to the small number of studies for each variable above, a more detailed classification of these variables for subgroup analyses is not feasible. Last, the study was limited to trials reported in the English language which limits the generalizability of the conclusion drawn from the study.

Light therapy could be useful for cancer patients experiencing sleep problems in clinical practices and it is safe, easy to deliver, and low-cost. However, well-designed large-scale RCT studies are needed to determine more accurately the treatment effects of light therapy on sleep quality in cancer patients. For future studies, there should include more diverse participants regarding race, and ethnic group as well as more specific types and stages of cancer. In addition, future studies could also investigate the effect of different phototherapy intensities (the cut-off points should be based on standard protocols) and different devices, which are shown to be lacking in the current research. Last but not least, future studies could apply cost-effectiveness analyses as part of the outcome measures.

## 5. Conclusion

Light therapy has the potential to improve sleep quality and support mental health in cancer patients. However, this systematic review and meta-analysis found no evidence regarding the effects of light therapy on some physiological sleep indexes. Additionally, due to the heterogeneity, and small sample size of included RCT studies, future trials need to consider using a larger sample, different characteristics of participants, phototherapy intensities and devices, longer intervention, and follow-up durations, to obtain a more accurate estimation of the benefit of light therapy in sleep quality for cancer patients.

## Data availability statement

The original contributions presented in the study are included in the article/Supplementary material, further inquiries can be directed to the corresponding author.

## Author contributions

LY and ZZ conducted the literature review, wrote and revised the manuscript. LY performed the statistical analysis. LL formulated the study objectives, conceptualized the study plan, supervised LY, and reviewed and edited the manuscript. All authors have read and approved the article.

## Conflict of interest

The authors declare that the research was conducted in the absence of any commercial or financial relationships that could be construed as a potential conflict of interest.

## Publisher’s note

All claims expressed in this article are solely those of the authors and do not necessarily represent those of their affiliated organizations, or those of the publisher, the editors and the reviewers. Any product that may be evaluated in this article, or claim that may be made by its manufacturer, is not guaranteed or endorsed by the publisher.
