# Supplementary material for: The effect of light therapy on sleep quality in cancer patients: a systematic review and meta-analysis of randomized controlled trials
Source: Front Psychiatry. 2023 Jul 10;14:1211561. doi: 10.3389/fpsyt.2023.1211561 (PMC10363736; doi:10.3389/fpsyt.2023.1211561)
Supplement: Supplementary file 1 [file Data_Sheet_1.docx]

Supplementary Material

The Effect of Light Therapy on Sleep Quality in Cancer Patients: A Systematic Review and Meta-Analysis of Randomized Controlled Trials

Liqing Yao, Zhiyi Zhang, and Lawrence T Lam*

*** Correspondence:** Lawrence T Lam: [tmlam@must.edu.mo](mailto:tmlam@must.edu.mo)

# Supplementary Tables

**Table S1.** Data bases and search strategy.

**Cochrane library**

| Searches | Results | Type |
| --- | --- | --- |
| MeSH descriptor: [Phototherapy] explode all trees or Phototherapies or Therapy, Photoradiation or Photoradiation Therapies or Therapies, Photoradiation or Light Therapy or Light Therapies or Therapies, Light or Therapy, Light or Photoradiation Therapy  **AND**  MeSH descriptor: [Neoplasms] explode all trees Tumor or Neoplasm or Tumors or Neoplasia or Neoplasias or Cancer or Cancers or Malignant Neoplasm or Malignancy or Malignancies or Malignant Neoplasms or Neoplasm, Malignant or Neoplasms, Malignant or Benign Neoplasms or Benign Neoplasm or Neoplasms, Benign or Neoplasm, Benign  **AND**  MeSH descriptor: [Sleep Quality] explode all trees or Qualities, Sleep or Quality, Sleep or Sleep Qualities | 73 | All fields |

**Embase**

| Searches | Results | Type |
| --- | --- | --- |
| (('neoplasms'/exp OR neoplasms) OR tumor OR neoplasm OR tumors OR neoplasia OR neoplasias OR cancer OR cancers OR (malignant AND neoplasm) OR malignancy OR malignancies OR malignant neoplasms OR (neoplasm, AND malignant) OR (neoplasms, AND malignant) OR benign neoplasms OR (benign AND neoplasm) OR (neoplasms, AND benign) OR (neoplasm, AND benign))  **AND**  ('phototherapy'/exp OR phototherapies OR (therapy, AND photoradiation) OR (photoradiation AND therapies) OR (therapies, AND photoradiation) OR (light AND therapy) OR (light AND therapies) OR (therapies, AND light) OR (therapy, AND light) OR (photoradiation AND therapy))  **AND**  ('sleep quality'/exp OR (qualities, AND sleep) OR (quality, AND sleep) OR (sleep AND qualities)) | 237 | All fields |

**Psyclnfo**

| Searches | Results | Type |
| --- | --- | --- |
| TX neoplasms OR TX  tumor OR TX neoplasia  OR TX cancer OR TX  malignant neoplasm OR  TX malignancy OR TX  neoplasms,malignant OR  TX benign neoplasm OR  TX neoplasms,benign  **AND**  TX phototherapy OR TX  light therapy  **AND**  TX sleep quality OR TX  quality of sleep OR TX  sleep qualities | 9 | All text |

**Pubmed**

| Searches | Results | Type |
| --- | --- | --- |
| (((((((((((Phototherapy[MeSH Terms]) OR (Phototherapies[All Fields])) OR (Therapy, Photoradiation[All Fields])) OR (Photoradiation Therapies[All Fields])) OR (Therapies, Photoradiation[All Fields])) OR (Light Therapy[All Fields])) OR (Light Therapies[All Fields])) OR (Therapies, Light[All Fields])) OR (Therapy, Light[All Fields])) OR (Photoradiation Therapy[All Fields]))  **AND** ((((((((((((((((((Neoplasms[MeSH Terms]) OR (Tumor[All Fields])) OR (Neoplasm[All Fields])) OR (Tumors[All Fields])) OR (Neoplasia[All Fields])) OR (Neoplasias[All Fields])) OR (Cancer[All Fields])) OR (Cancers[All Fields])) OR (Malignant Neoplasm[All Fields])) OR (Malignancy[All Fields])) OR (Malignancies[All Fields])) OR (Malignant Neoplasms[All Fields])) OR (Neoplasm, Malignant[All Fields])) OR (Neoplasms, Malignant[All Fields])) OR (Benign Neoplasms[All Fields])) OR (Benign Neoplasm[All Fields])) OR (Neoplasms, Benign[All Fields])) OR (Neoplasm, Benign[All Fields])))  **AND**  (((("Sleep Quality"[Mesh]) OR (Qualities, Sleep)) OR (Quality, Sleep)) OR (Sleep Qualities)) | 70 | All fields |

**Scopus**

| Searches | Results | Type |
| --- | --- | --- |
| ((TITLE-ABS-KEY (phototherapy) OR TITLE-ABS-KEY (phototherapies) OR TITLE-ABS-KEY (therapy, AND  photoradiation)  OR  TITLE-ABS-KEY (photoradiation  AND  therapies) OR  TITLE-ABS-KEY(therapies,  AND  photoradiation)  OR  TITLE-ABS-KEY (light  AND  therapy)  OR  TITLE-ABS-KEY (light  AND  therapies)  OR  TITLE-ABS-KEY (therapies,  AND  light)  OR  TITLE-ABS-KEY (therapy,  AND  light)  OR  TITLE-ABS-KEY (photoradiation  AND  therapy)))  **AND**  ((TITLE-ABS-KEY (sleep AND quality) OR TITLE-ABS-KEY (qualities, AND sleep) OR TITLE-ABS-KEY (quality, AND sleep) OR TITLE-ABS-KEY (sleep AND qualities)))  **AND**  ( ( TITLE-ABS-KEY ( neoplasms )  OR  TITLE-ABS-KEY ( tumor )  OR  TITLE-ABS-KEY ( neoplasm )  OR  TITLE-ABS-KEY ( tumors )  OR  TITLE-ABS-KEY ( neoplasia )  OR  TITLE-ABS-KEY ( neoplasias )  OR  TITLE-ABS-KEY ( cancer )  OR  TITLE-ABS-KEY ( cancers )  OR  TITLE-ABS-KEY ( malignant  AND neoplasm )  OR  TITLE-ABS-KEY ( malignancy )  OR  TITLE-ABS-KEY ( malignancies )  OR  TITLE-ABS-KEY ( malignant neoplasms )  OR  TITLE-ABS-KEY ( neoplasm,  AND malignant )  OR  TITLE-ABS-KEY ( neoplasms,  AND malignant )  OR  TITLE-ABS-KEY ( benign neoplasms )  OR  TITLE-ABS-KEY ( benign  AND neoplasm )  OR  TITLE-ABS-KEY ( neoplasms,  AND benign )  OR  TITLE-ABS-KEY ( neoplasm,  AND benign ) ) ) | 126 | Title Abstract Keyword |

**Web of science**

| Searches | Results | Type |
| --- | --- | --- |
| (TS=(Phototherapy)) OR TS= (Phototherapies or Therapy, Photoradiation or Photoradiation Therapies or Therapies, Photoradiation or LightTherapy or Light Therapies or Therapies, Light or Therapy, Light or PhotoradiationTherapy)  **AND**  (TS=(Neoplasms)) OR TS = (Tumor or Neoplasm or Tumors or Neoplasia or Neoplasias or Cancer or Cancers or Malignant Neoplasm or Malignancy or Malignanciesor Malignant Neoplasms or Neoplasm, Malignant or Neoplasms, Malignant or Benign Neoplasms or Benign Neoplasm or Neoplasms, Benign or Neoplasm, Benign)  **AND**  (TS= (Sleep Quality)) OR TS= (Qualities, Sleep or Quality, Sleep or Sleep Qualities) | 82 | Topic |
